# Supplementary material for: The Combination of Anti-CD47 Antibody with CTLA4 Blockade Enhances Anti-Tumor Immunity in Non-Small Cell Lung Cancer via Normalization of Tumor Vasculature and Reprogramming of the Immune Microenvironment
Source: Cancers (Basel). 2024 Feb 19;16(4):832. doi: 10.3390/cancers16040832 (PMC10887353; doi:10.3390/cancers16040832)
Supplement: Supplementary file 1 [file cancers-16-00832-s001.zip › cancers-2669656-figures.pdf]

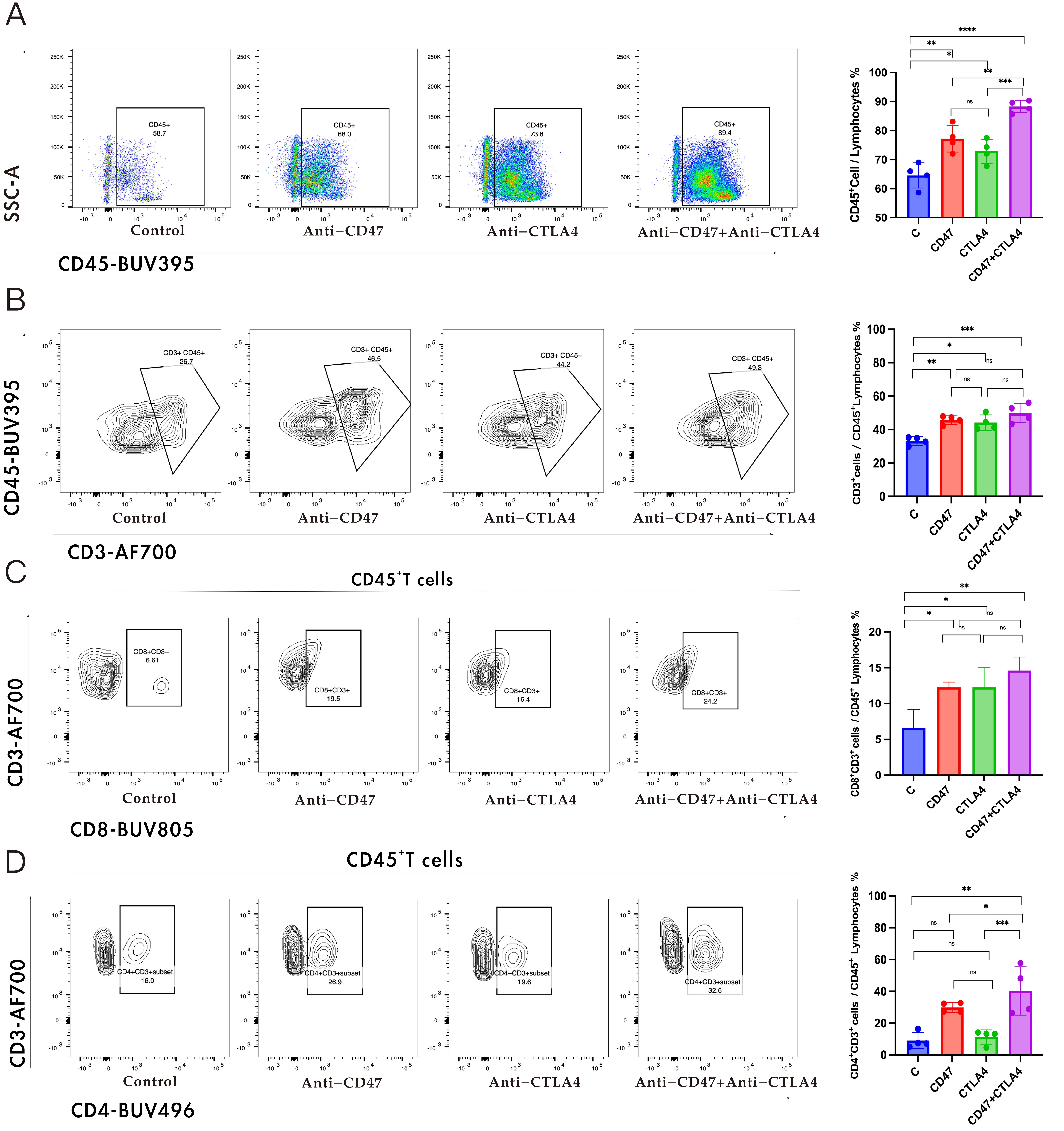

A
SSC-A
CD45+ 58.7
CD45+ 68.0
CD45+ 73.6
CD45+ 89.4
Control
Anti−CD47
Anti−CTLA4
Anti−CD47+Anti−CTLA4
CD45-BUV395
CD45⁺Cell / Lymphocytes %
C
CD47
CTLA4
CD47+CTLA4
B
CD45-BUV395
CD3+ CD45+ 26.7
CD3+ CD45+ 46.5
CD3+ CD45+ 44.2
CD3+ CD45+ 49.3
Control
Anti−CD47
Anti−CTLA4
Anti−CD47+Anti−CTLA4
CD3-AF700
CD3⁺cells / CD45⁺Lymphocytes %
C
CD45⁺T cells
CD3-AF700
CD8+CD3+ 6.61
CD8+CD3+ 19.5
CD8+CD3+ 16.4
CD8+CD3+ 24.2
Control
Anti−CD47
Anti−CTLA4
Anti−CD47+Anti−CTLA4
CD8-BUV805
CD8⁺CD3⁺ cells / CD45⁺ Lymphocytes %
D
CD45⁺T cells
CD3-AF700
CD4+CD3+subset 16.0
CD4+CD3+subset 26.9
CD4+CD3+subset 19.6
CD4+CD3+subset 32.6
Control
Anti−CD47
Anti−CTLA4
Anti−CD47+Anti−CTLA4
CD4-BUV496
CD4⁺CD3⁺ cells / CD45⁺ Lymphocytes %

Figure S1. Changes in fractions of $CD45^+$, $CD3^+$, or $CD8^+$ T cells in non-small cell lung cancer (NSCLC) after anti-CD47 antibody (Ab) or anti-CTLA4 Ab (i.e., an immune checkpoint blocker) alone or in combination. Lewis lung carcinoma (LLC) cells were implanted in C57BL/6 mice treated with control IgG, anti-CD47 Ab, and anti-CTLA4 Ab, alone or in combination. Flow cytometric cell sorting at day 24 revealed the following: (A) Both monotherapy and combination therapy promote intra-tumoral infiltration of $CD45^+$ cells, with the combination therapy showing the most significant effect. (B) The fraction of $CD3^+$ T cells increases after anti-CD47 Ab blockade, anti-CTLA4 Ab blockade, or their combination therapy compared to control IgG. (C–D) Combination therapy significantly promotes intra-tumoral infiltration of $CD8^+$ T cells and $CD4^+$ T cells compared to other groups. Data in (A–D) are analyzed with a one-way analysis of variance. *$p < 0.05$, **$p < 0.01$, and ***$p < 0.001$; ns, not significant.

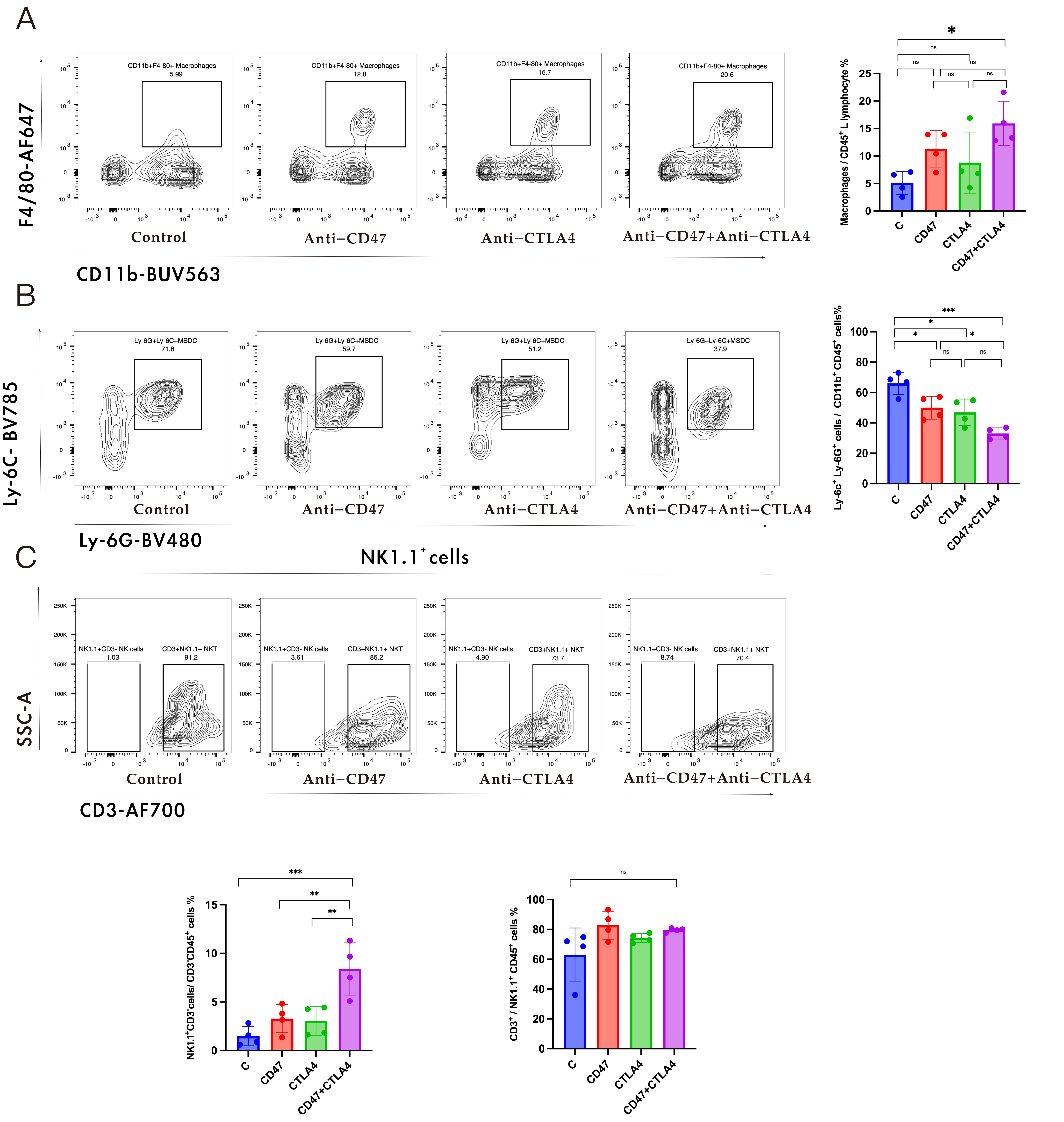
A
F4/80-AF647
CD11b+F4-80+ Macrophages
5.99
CD11b+F4-80+ Macrophages
12.8
CD11b+F4-80+ Macrophages
15.7
CD11b+F4-80+ Macrophages
20.6
Control
Anti−CD47
Anti−CTLA4
Anti−CD47+Anti−CTLA4
CD11b-BUV563
Macrophages / CD45+ L lymphocyte %
C
CD47
CTLA4
CD47+CTLA4
*
ns
B
Ly-6C- BV785
Ly-6G+Ly-6C+MSDC
71.8
Ly-6G+Ly-6C+MSDC
59.7
Ly-6G+Ly-6C+MSDC
51.2
Ly-6G+Ly-6C+MSDC
37.9
Control
Anti−CD47
Anti−CTLA4
Anti−CD47+Anti−CTLA4
Ly-6G-BV480
Ly-6c+ Ly-6G+ cells / CD11b+ CD45+ cells%
***
*
ns
C
CD47
CTLA4
CD47+CTLA4
C
NK1.1+ cells
SSC-A
NK1.1+CD3- NK cells
1.03
CD3+NK1.1+ NKT
91.2
NK1.1+CD3- NK cells
3.61
CD3+NK1.1+ NKT
85.2
NK1.1+CD3- NK cells
4.90
CD3+NK1.1+ NKT
73.7
NK1.1+CD3- NK cells
8.74
CD3+NK1.1+ NKT
70.4
Control
Anti−CD47
Anti−CTLA4
Anti−CD47+Anti−CTLA4
CD3-AF700
NK1.1+CD3-cells/ CD3-CD45+ cells %
***
**
C
CD47
CTLA4
CD47+CTLA4
CD3+ / NK1.1+ CD45+ cells %
ns
C
CD47
CTLA4
CD47+CTLA4

Figure S2. Changes of macrophages, myeloid-derived suppressor cells (MDSC), natural killer (NK) cells, and natural killer T (NKT) cells in tumor infiltration in NSCLC after anti-CD47 antibody (Ab) or anti-CTLA4 Ab (i.e., an immune checkpoint blocker) alone or in combination. (A) Lewis lung carcinoma (LLC) cells were implanted in C57BL/6 mice treated with control IgG, anti-CD47 Ab, and anti-CTLA4 Ab, alone or in combination. Flow cytometric cell sorting at day 24 revealed the following: Compared to control IgG, the fraction of tumor-associated macrophages (TAMs) is increased after combination therapy. (B) Combination therapy decreases the fraction of myeloid-derived suppressor cells (MDSCs). (C) The combination therapy promotes the infiltration of NK cells in the tumor compared to single-agent therapy and the control group, but no significant change is observed in NKT cells. Data are expressed as mean ± standard error (SE). Data in (A–C) are analyzed with a one-way analysis of variance. $^{*}p < 0.05$, $^{**}p < 0.01$, and $^{***}p < 0.001$; ns, not significant.

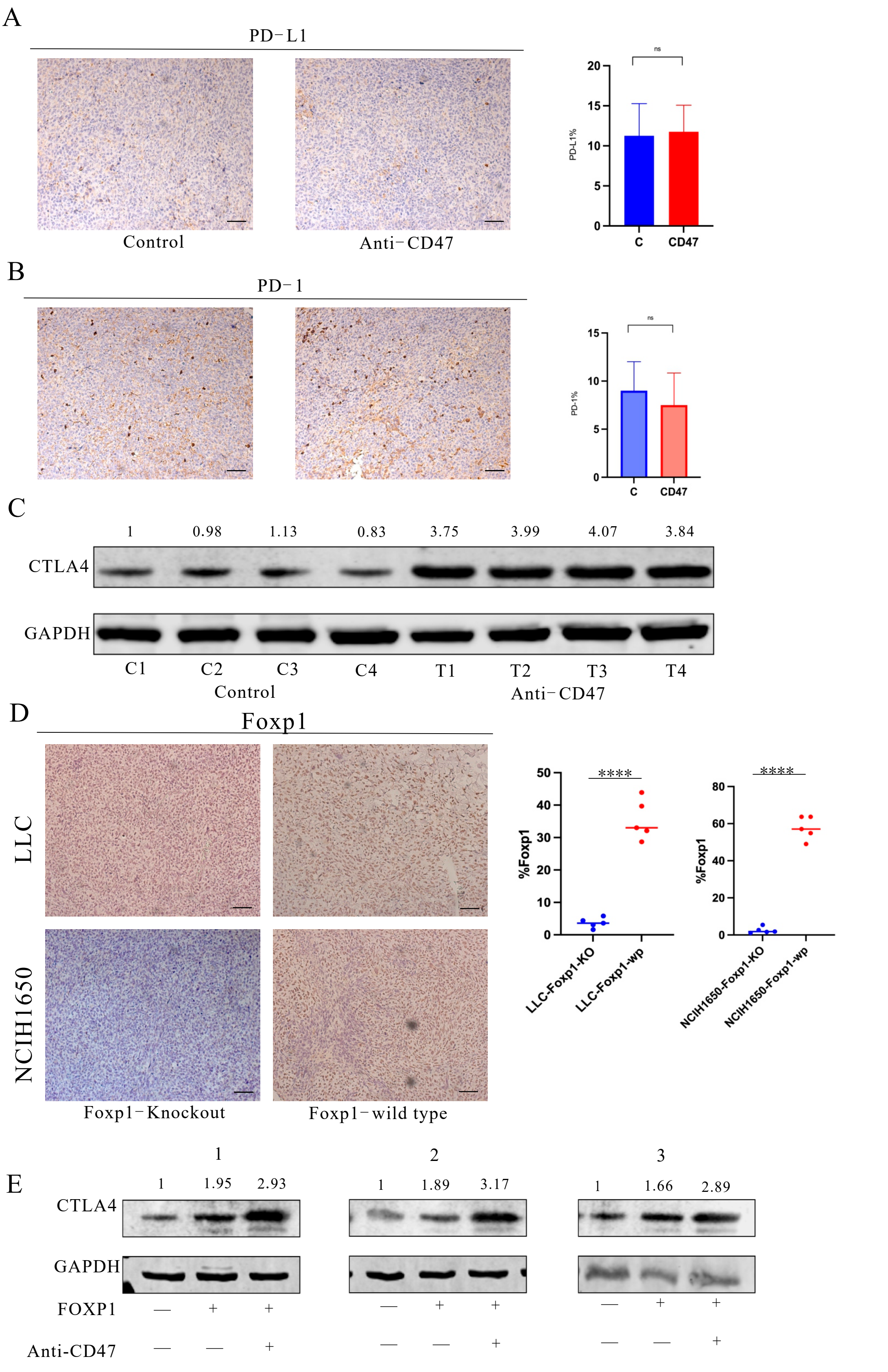

Figure S3. The protein level of PD-L1/PD1 in tumor tissues of Lewis lung carcinoma (LLC) cells of NSCLC-bearing mice after anti-CD47 antibody (Ab) therapy. (A–B) Representative

immunohistochemistry images and quantitative analysis showing no significant change in PD-L1 and PD-1 levels in tumor tissues after anti-CD47 Ab therapy. (C) Measurement of CTLA4 level in tumor tissues using immunoblots after treatment with anti-CD47 Ab or control IgG. (D) IHC assesses Foxp1 level in NSCLC-bearing mice with Foxp1 deletion.The foxp1 level were significantly decreased. (E) Foxp1 deletion on LLC partially prevents the increase in CTLA4 level induced by anti-CD47 Ab treatment. Data are expressed as mean ± standard error (SE). Data in (A–E) are evaluated with unpaired Student's t-test. *p < 0.05, **p < 0.01, and ***p < 0.001; ns, not significant.

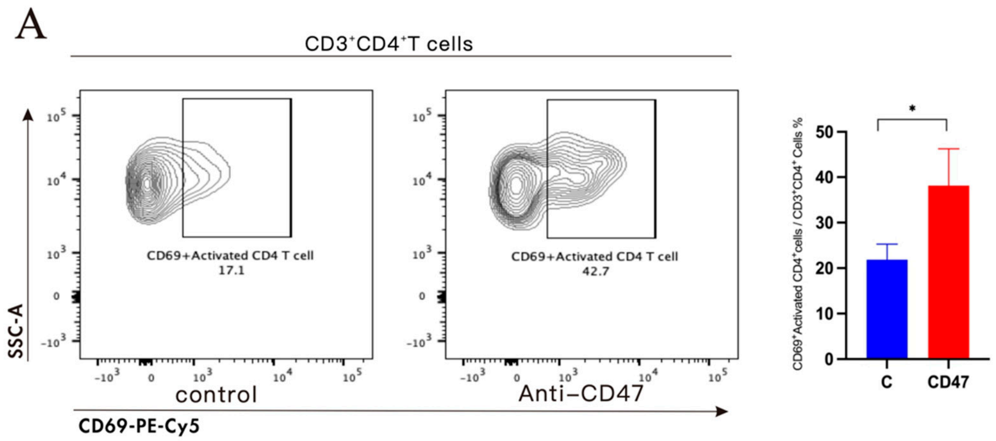

Figure S4. Anti-CD47 antibody (Ab) increases the fraction activated CD4⁺ T cells among immune cells infiltrating tumors of Lewis lung carcinoma cells. (A) Anti-CD47 Ab therapy promotes the fraction of activated CD69⁺ CD4⁺ T cells. Data are expressed as mean ± standard error (SE). Data in (A) are evaluated with unpaired Student's t-test. *p < 0.05, **p < 0.01, and ***p < 0.001; ns, not significant.

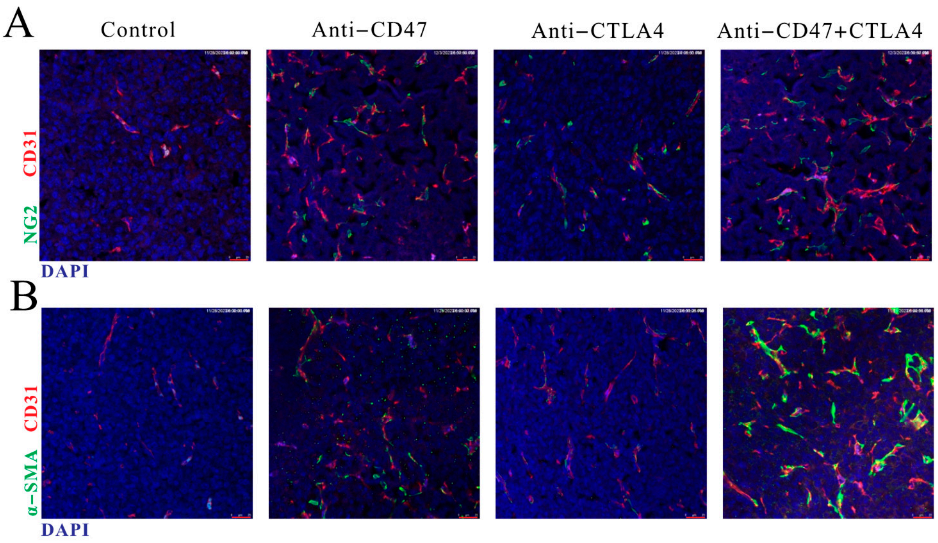

Figure S5. The effect of combination therapy on tumor vascular normalization.

Mice (n = 8) bearing tumors of subcutaneously transplanted LLC cells were treated with anti-CD47 Ab thrice weekly until tumors became palpable (60–80 mm^3). Data are representative of at least three independent experiments. (A–B) Tumor tissues of LLC cells were collected on day 24 after treatment with anti-CD47 Ab, anti-CTLA4 Ab or anti-CD47 Ab+ anti-CTLA4 Ab . Blood vessel number within tumors as determined using CD31 staining (A-B). Images were obtained for blood vessels expressing NG2 (A) and α-SMA (B) in tumor tissues to evaluate blood vessels covered by pericytes and wall cells. Data are representative of three independent experiments. Scale bars, 50 μm. DAPI, 4′,6-diamidino-2-phenylindole. Data are expressed as mean ± standard error (SE). Data in (A–B) are evaluated with unpaired Student's t-test. *$p < 0.05$, **$p < 0.01$, and ***$p < 0.001$; ns, not significant.
