# Supplementary material for: The Combination of Anti-CD47 Antibody with CTLA4 Blockade Enhances Anti-Tumor Immunity in Non-Small Cell Lung Cancer via Normalization of Tumor Vasculature and Reprogramming of the Immune Microenvironment
Source: Cancers (Basel). 2024 Feb 19;16(4):832. doi: 10.3390/cancers16040832 (PMC10887353; doi:10.3390/cancers16040832)

Figure 3D

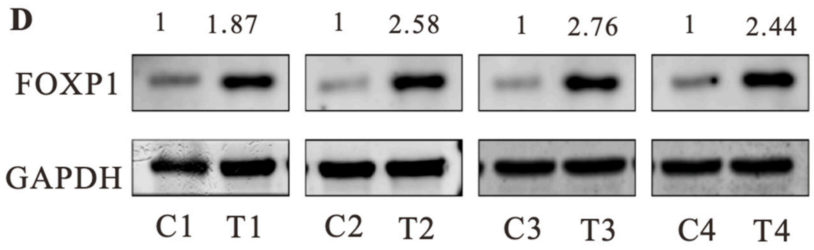

Foxp1: 75 kDa

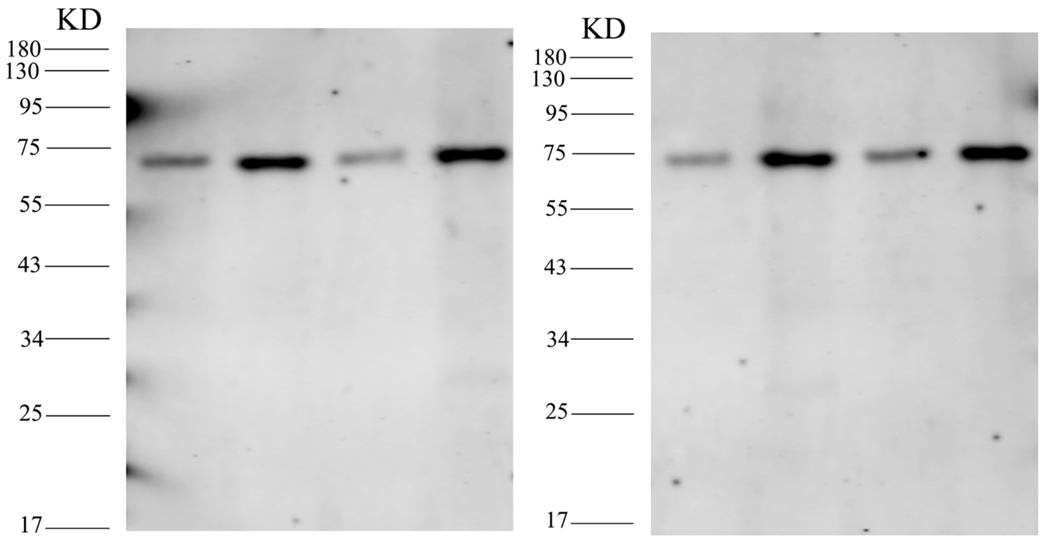

GAPDH：37kDa

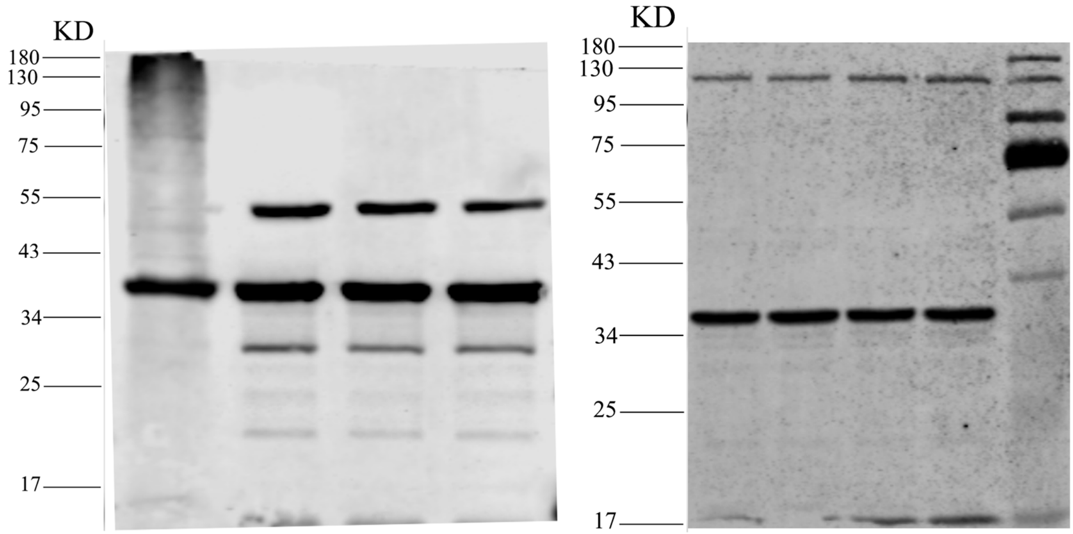

Figure 3E

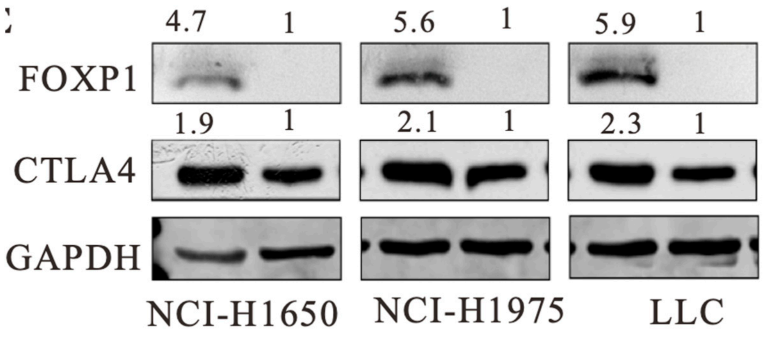

Foxp1:75kDa

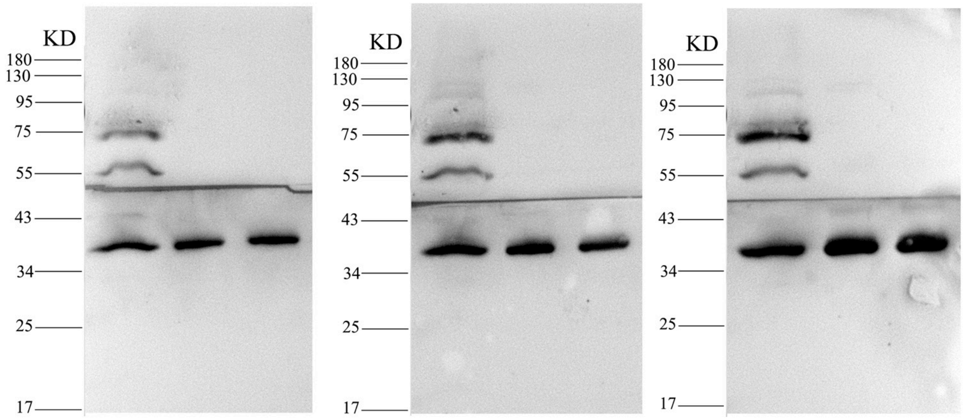

CTLA4：37kDa

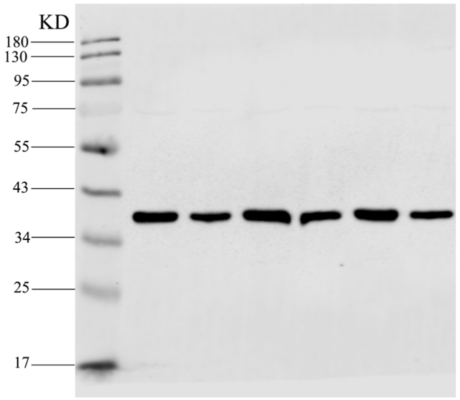

GAPDH：37kDa

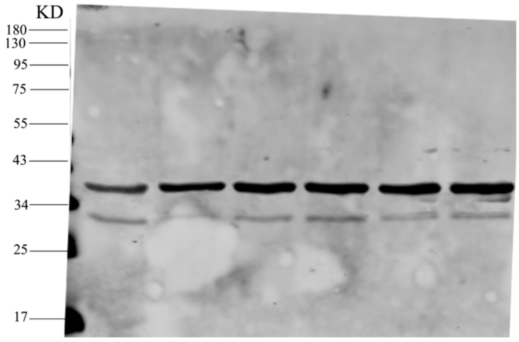

Supplement Figure S3C

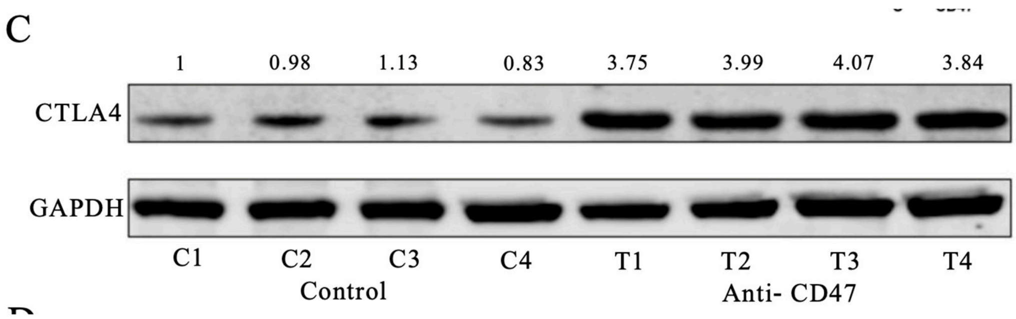

CTLA4：37kDa

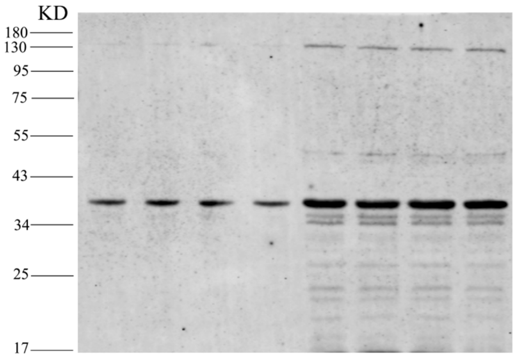

GAPDH：37kDa

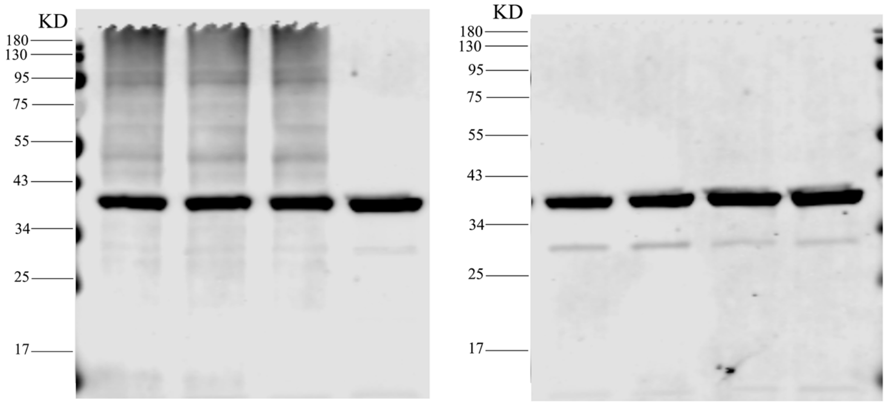

Supplement Figure S3E

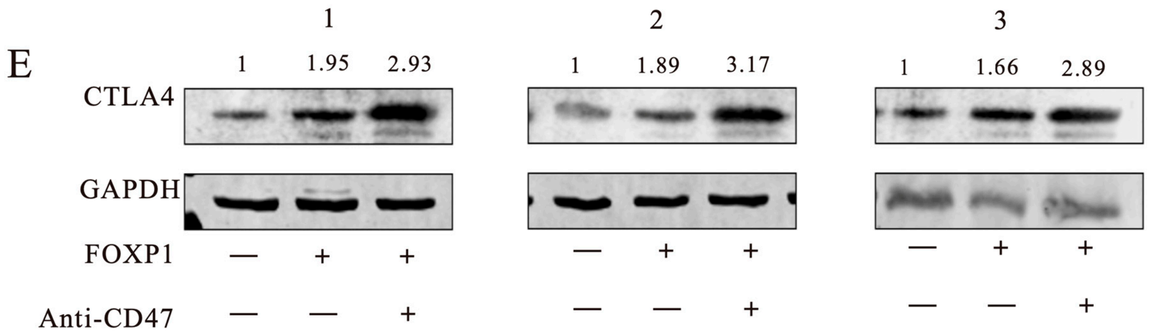

CTLA4：37kDa

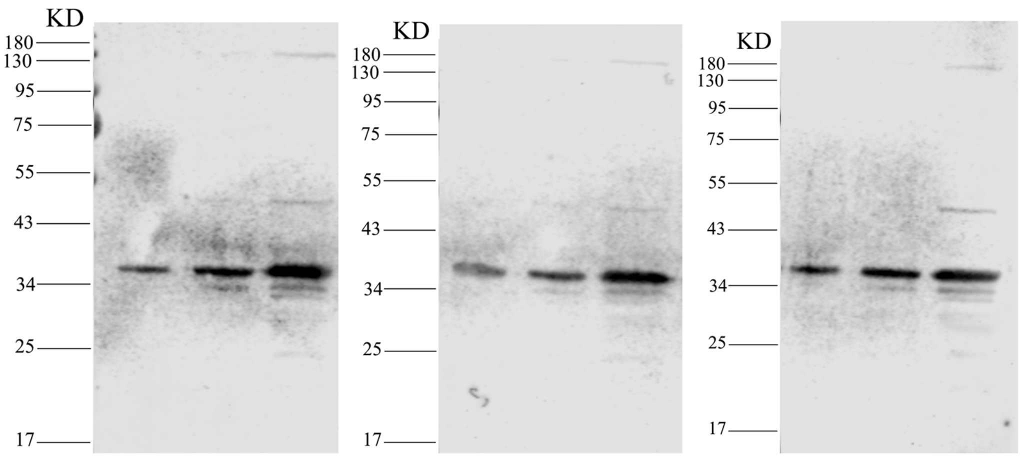

GAPDH：37kDa

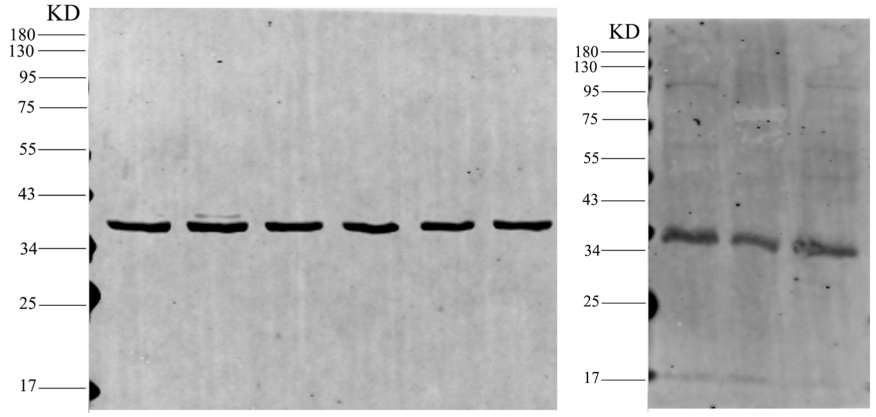

Supplement: Supplementary file 1 [file cancers-16-00832-s001.zip › cancers-2669656-File S1.pdf]
